# From Waste Vegetable Oil to a Green Compatibilizer for HDPE/PA6 Blends

**DOI:** 10.3390/polym15204178

**Published:** 2023-10-21

**Authors:** Miriam Cappello, Giovanna Strangis, Patrizia Cinelli, Caterina Camodeca, Sara Filippi, Giovanni Polacco, Maurizia Seggiani

**Affiliations:** 1 Department of Civil and Industrial Engineering, University of Pisa, L.go L. Lazzarino 2, 56122 Pisa, Italy; miriam.cappello@unipi.it (M.C.); giovanna.strangis@phd.unipi.it (G.S.); patrizia.cinelli@unipi.it (P.C.); maurizia.seggiani@unipi.it (M.S.); 2 Department of Pharmacy, University of Pisa, Via Bonanno Pisano 33, 56126 Pisa, Italy; caterina.camodeca@unipi.it

**Keywords:** high-density polyethylene (HDPE), polyamide-6 (PA6), waste vegetable oil, compatibilizer precursor, polymer blends

## Abstract

When properly compatibilized, the blending of polyethylene (PE) and polyamide (PA) leads to materials that combine low prices, suitable processability, impact resistance, and attractive mechanical properties. Moreover, the possibility of using these polymers without prior separation may be a suitable opportunity for their recycling. In this work, the use of an epoxidized waste vegetable oil (EWVO) was investigated as a green compatibilizer precursor (CP) for the reactive blending of a high-density PE (HDPE) with a polyamide-6 (PA6). EWVO was synthesized from waste vegetable cooking oil (WVO) using ion-exchange resin (Amberlite) as a heterogeneous catalyst. HDPE/PA6 blends were produced with different weight ratios (25/75, 75/25, 85/15) and amounts of EWVO (1, 2, 5 phr). Samples with WVO or a commercial fossil-based CP were also prepared for comparison. All the blends were characterized by scanning electron microscopy (SEM), differential scanning calorimetry (DSC), rheology, and mechanical tests. In the case of HDPE/PA6 75/25 and 85/15 blends, the addition of EWVO at 2 phr showed a satisfactory compatibilizing effect, thus yielding a material with improved mechanical properties with respect to the blend without compatibilizer. On the contrary, the HDPE/PA6 25/75 ratio yielded a material with a high degree of crosslinking that could not be further processed or characterized. In conclusion, the results showed that EWVO had a suitable compatibilizing effect in HDPE/PA6 blends with high HDPE content, while it resulted in unsuitable for blends with high content of PA6.

## 1. Introduction

The compounding of polyethylenes (PEs) and polyamides (PAs) attracted the interest of the scientific community several years ago with the idea of combining the properties of these two polymers to obtain low-price materials with suitable processability, impact, and mechanical properties and high oil resistance [1]. The high-density polyethylene and polyamide-6 blend system had great potential to be used, especially in high-barrier packaging applications where the polyethylene provides a high water vapor barrier, and the polyamide provides an oxygen barrier. Due to their incompatibility, in order to obtain the highest barrier efficiency, polyethylene and polyamide are usually processed to obtain multilayer films. When these multilayer films reach the end of their life, it is not possible to separate and recycle independently the different layers. However, a simple melting would lead to a coarse morphology that is not ideal for high-barrier packaging applications. Therefore, the use of a compatibilizer is necessary to reduce the interfacial tension between PEs and PAs and improve the quality of the recycled blends. Additionally, PEs and PAs can be found together in different plastic wastes. Therefore, an additional advantage of this blending is the possibility of direct processing without prior separation of the two components. This will result in recycling with reduced costs and environmental impact.

Of course, due to their different chemical composition and polarity, PEs and PAs are highly immiscible, and a simple melt blending under high shear conditions leads to a markedly biphasic material with poor mechanical properties. For this reason, the use of a compatibilizer as an additional component of the blend is mandatory. The most studied PE/PA compatibilizers are copolymers containing segments able to interact with both components. These copolymers can be synthesized separately and then added to the mixture or produced in situ by reactive blending. The latter solution involves the use of a polymer that is highly miscible with one of the two components but contains functional groups able to chemically react with the second one. The result is a graft copolymer that maintains the original main chain, with the second polymer located in covalently bonded branches. For this reason, the functionalized polymer is defined as the compatibilizer precursor (CP), the real compatibilizer of the reaction product. If the CP is added to the mixture and the chemical reaction takes place during the mixing step, then the compatibilizer is formed in situ and is intrinsically located at the interface between the two components. In the case of PE and PA, since the polyamide contains amine and carboxylic terminal groups, the CP is usually a polyethylene-compatible polymer that is able to react with those groups. The most popular examples are polyolefins functionalized with maleic anhydride, acrylic acid, isocyanate, epoxy, oxazoline, or glycidyl methacrylate [1,2,3,4,5,6,7,8,9]. Of course, the type of functional groups present in the CP influences its reactivity toward PA and, thus, its effect in the subsequent compatibilization step. Filippi et al. [2] and Jiang et al. [3] showed that copolymers functionalized with acrylic acid or maleic anhydride react with amine groups of PA, thus forming a grafted copolymer (CP-*g*-PA), but no reaction occurred between the compatibilizer and the carboxyl groups [2,3]. On the contrary, CPs containing glycidyl methacrylate groups are able to react with both the amine and carboxyl groups of the PA, as reported by Chiono et al. [4]. In addition to polyolefins, other options have been considered as CP for the PE/PA system. Oshinki et al. [10,11,12] and Takeda et al. [13] investigated the behavior of SEBS-g-MA as an additive for different kinds of PAs, such as PA6, PA11, PA12, PA4,6, PA6,6, PA6,9, PA6,12. The authors observed that the dispersed particles were smaller in the case of PAx polymers than in PAx,y ones. The different morphology was attributed to the different end groups of the two kinds of polymers: PAx has amine and carboxyl functionalities at the end of the chains, while PAx,y has only NH_2_ as the end group [13].

Moreover, many authors reported the influence of other factors, such as the amount and molecular weight of CP, as well as its content of functional groups, on the effectiveness of compatibilization. The latter can be qualitatively evaluated through direct observation of the dimension, shape, and distribution of the dispersed phase, which are strictly connected to the quantitative effect on the final mechanical and rheological properties of the blends [2,6,9,13,14,15,16,17,18,19,20,21].

In recent years, epoxidized vegetable oils (EVO) [22] have gained attention in the polymer field due to their potential use in many applications. A few examples are the use of EVO to substitute phthalates as a plasticizer in PVC [23] or as a reactive diluent in epoxy resins [24,25], coatings [26], thermosets, and composites [27,28]. The interested reader can find an exhaustive description of these applications in recent reviews like those by Thomas and Patil [29] and by Chong et al. [30]. Of course, due to the presence of epoxy groups, EVO has also been used as compatibilizers, like in polylactic acid composites [31,32,33,34].

In this context, the present study aims to investigate epoxidized waste vegetable oil (EWVO) as a potential compatibilizer precursor for high-density polyethylene (HDPE) and polyamide-6 (PA6) blends. For this purpose, waste vegetable cooking oil (WVO) was chosen as the raw material in order to reduce the environmental impact and costs of the process. The oil was first functionalized by an epoxidation reaction to convert it into a low-molecular-weight CP able to react with the terminal groups of the polyamide. Blends with different HDPE/PA6 weight ratios were produced by melt mixing, adding different amounts of EWVO. The blends were then characterized from a morphological, thermal, and mechanical point of view in order to evaluate the compatibilizing effect of the synthesized CP. A non-functionalized WVO and a commercial block copolymer containing epoxy groups (Lotader AX8840) were also used for comparison. The novelty of the work consists of the use of a CP with a low molecular weight, coming from the waste bio-based source, as an alternative to the above-mentioned functionalized fossil-based copolymers commonly used for PE/PA blends.

## 2. Materials and Methods

### 2.1. Materials

The blends were prepared using the following polymers: a commercial HDPE sample, Eltex A4009MFN1325 (Ineos Manufacturing Italia SpA, Rosignano Solvay, Italy), and a commercial PA6, kindly furnished by Snia Tecnopolimeri. HDPE has a melt flow index (MFI) of 0.9 g/10 min (190 °C/2.16 kg ISO 1133 [35]); PA6 had a relative viscosity in sulfuric acid (95.7%) of 3.66; and the contents of amine and carboxylic end groups were equal to 34 and 35 meq∙kg^−1^, respectively. The bio-based compatibilizer precursor (CP) was synthesized using the following raw materials: a waste vegetable cooking oil (WVO), furnished by the Physis srl (Pisa, Italy); glacial acetic acid (GAA) (99.8%, Sigma-Aldrich, St. Louis, MO, USA); hydrogen peroxide (35%, Sigma-Aldrich); and Ion Exchanger Amberlite^®^ IR-120 (Supelco, St. Louis, MO, USA).

A commercial ethylene–glycidylmethacrylate (EGMA) copolymer (Lotader GMA AX8840), provided by Elf-Atochem (Alessandria, Italy), containing 8 wt.% of GMA and an MFI equal to 5.0g/10 min (190 °C/2.16 kg ISO 1133), was used for comparison.

### 2.2. Determination of Molecular Weight (Mw) and C=C Content of WVO

The unsaturation degree of oils is usually expressed by the iodine value (IV) because it was traditionally determined by indirect titration of double bonds involving I_2_ determination. However, Miyake et al. [36] developed a method to determine by ^1^H-NMR spectroscopy both the content of double bonds and the molecular weight of vegetable oils. Based on this method, the average number of double-bonded protons and molecular weight of the oil is provided by Equations (1) and (2), respectively:

(1) $${number}_{C=C}=\frac{1}{2}\cdot \left(\frac{a+b-c/4}{A_{p}}\right)$$

(2) $$M_{w}=\frac{15.034}{3}\cdot \left(\frac{i+j}{A_{p}}\right)+\frac{14.026}{2}\cdot \left(\frac{d+e+f+g+h}{A_{p}}\right)+\frac{173.100}{4}\cdot \left(\frac{c}{A_{p}}\right)+\frac{26.016}{2}\cdot \left(\frac{a+b-c/4}{A_{p}}\right)$$

where *a*–*j* are the areas of the signals defined in Table 1 and $A_{p}$ is the area per proton that is equal to 1 when the signal’s areas are normalized; otherwise, it can be calculated by dividing the area of the signal *c* by 4, i.e., the number of the protons in the methylene of the glyceryl group.

The ^1^H-NMR spectra were recorded on a Bruker Avance III HD 400 MHz spectrometer (Billerica, MA, USA). Chemical shifts (δ) are reported in parts per million.

### 2.3. Compatibilizer Precursor (CP) Synthesis

The EWVO was synthesized using a procedure taken from the literature [37]: about 100 g (0.115 mol) of WVO and 15 g of catalyst (Amberlite^®^ IR-120, 15% by weight of WVO) were put into a 500 mL round-bottom flask equipped with a reflux condenser, a dropping funnel, and a mechanical stirrer. The dropping funnel was filled with a mix containing 11.2 g of glacial acetic acid and 72.2 g of hydrogen peroxide (35 wt.%). The amount of glacial acetic acid and H_2_O_2_ were calculated to obtain a molar ratio of double bonds, glacial acetic acid, and H_2_O_2_ equal to 1:0.5:2. The system was heated, and when it reached 70 °C, the mix was added, drop by drop, in 30 min. The reaction was performed at 70 °C for 6.5 h, under stirring. The resulting mixture was neutralized with an aqueous solution of 5 wt.% of NaHCO_3,_ and chloroform was added to extract the desired product. The organic and aqueous phases were separated. The aqueous phase was washed with chloroform, subsequently recovered, and added to the organic phase. The latter was washed three times with deionized water and distilled under vacuum to remove the solvent. Samples of the mixture were taken after 2.5, 4.0, and 6.5 h and analyzed by Fourier transform infrared (FT-IR) spectroscopy, carried out by a Spectrum 400 Spectrometer (Perkin Elmer, Waltham, MA, USA).

### 2.4. Blends Preparation

All the blends were prepared by feeding the desired amounts of all the polymeric components into the 50 mL chamber of a Brabender Plasticorder static mixer (Brabender, GmbH & Co. KG., Duisburg, Germany). The chamber was preheated to 235 °C. WVO, or EWVO, was fed for approximately 2 min after the polymer components had melted. The complete feeding step was carried out in 3 min at a rotor speed of 30 rpm. After that, the rotor speed was increased to 60 rpm. The reactive blending was performed at 60 rpm and 235 °C for 10 min. The entire process was conducted under nitrogen. During the blending, the applied torque was measured and recorded.

Blends were prepared using the following HDPE/PA6 weight ratios: 85/15, 75/25, 25/75. Different amounts of EWVO (1, 2, 5 phr) were added, obtaining ternary blends HDPE/PA6/EWVO. Ternary systems containing WVO or Lotader in place of EWVO were also prepared for comparison. The prepared blends are listed in Table 2.

### 2.5. Blends Characterization

For scanning electron microscopy (SEM), a COXEM EM-30N SEM (COXEM Co., Ltd., Daejeo, Republic of Korea) was used to investigate the blend morphology. The analysis was conducted on fractured surfaces coated with gold.

Thermal properties were evaluated by a Perkin Elmer Differential Scanning Calorimeter (Pyris 1 DSC 6000, Waltham, MA, USA). Samples of about 10 mg were analyzed with in the temperature range of 30–250 °C. The procedure consisted of three steps: a first heating from 30 to 250 °C followed by an isothermal at 250 °C for 3 min to remove the thermal history of the sample; a cooling from 250 to 30 °C; and a second heating from 30 to 250 °C. The cooling and heating rate was 10 °C/min.

Frequency sweeps in the range of 0.05–100 Hz were performed at 240 °C using an MCR 92 Rheometer (Anton Paar, Rivoli, Italy) equipped with a plate–plate geometry to investigate the rheological behavior of the blends.

Mechanical properties were evaluated by tensile tests carried out on the ISO 527-1 [38] dog-bone specimens produced by a mini-injection molding press (ZWP Proma, Długołęka, Poland). The pellets were loaded at 235 °C and, after about 1 min, the melted materials were injected in a steel dog-bone mold (65 °C) with the following dimensions: overall length = 80 mm, gauge length = 25 mm, width = 4 mm, thickness = 2 mm. The tests were conducted at room temperature, with a test speed of 10 mm/min, using an MTS 50 KN apparatus (Eden Prairie, MN, USA) equipped with a 2 kN load cell. All tests were carried out on at least 5 samples to guarantee the reliability of the collected data.

## 3. Results and Discussion

### 3.1. Determination of Mw and C=C Content of WVO

**Figure 1:**
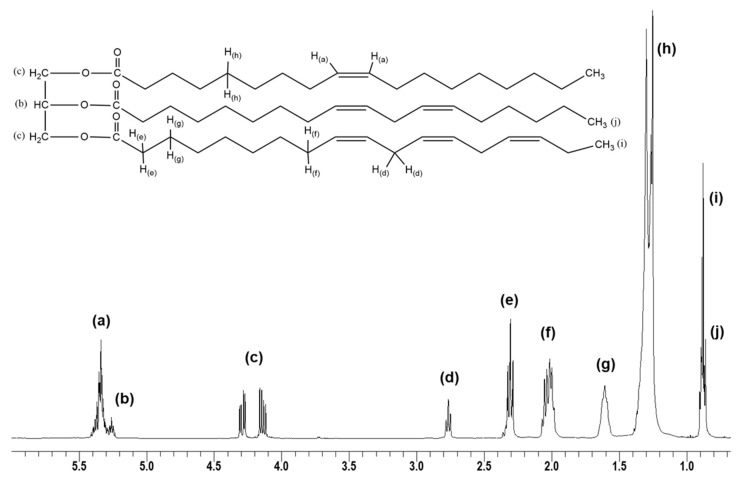
Chemical structure and ^1^H-NMR spectrum of WVO (letters as in Table 1).

Figure 1 and Table 3 report the ^1^H-NMR spectrum of WVO and the intensities of the signals, respectively. The latter values were used to calculate the molecular weight, *M_w_* (Equation (1)), and the content of double bonds (Equation (2)). The obtained values were 869 g/mol and 3.23, respectively.

The number of double bonds corresponds to the number of mols of C=C per mol of WVO. However, it is more common to refer to the mol of C=C per grams of vegetable oil. Therefore, for convenience, this value was calculated using Equation (3) and resulted in equal to 3.72 × 10^−3^ mol of C=C/g of oil.

(3) $${mol}_{C=C}=\frac{{number}_{C=C}}{M_{w,oil}}$$

### 3.2. Compatibilizer Precursor (CP)

**Figure 2:**
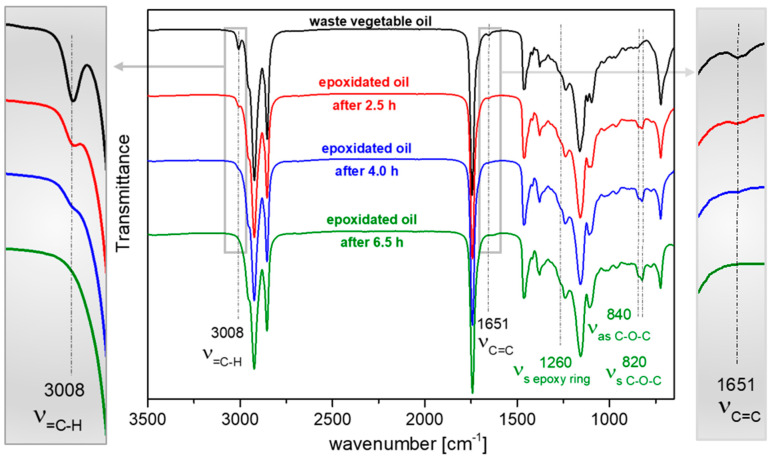
FT-IR spectra of samples of WVO (black) and epoxidation reaction mixture samples after 2.5 (red), 4.0 (blue), and 6.5 h (green).

The synthesis of EWVO was carried out by substituting the C=C double bonds in WVO with epoxy rings, and the reaction was monitored by FT-IR analysis (Figure 2). As can be noted, WVO showed two peaks at 3008 and 1651 cm^−1^, corresponding to the stretching of the unsaturated =C-H and C=C bonds, respectively [39]. As far as the reaction takes place, these two peaks showed a reduced intensity (see magnifications on the left and right-hand side of Figure 2), and there was a contemporary appearance of peaks at 1260, 840, and 820 cm^−1^, ascribed to the symmetrical stretching of C-O-C epoxy ring, and to the asymmetrical and symmetrical stretching of the C-O epoxy bonds, respectively. After 6.5 h of reaction, the peaks related to the double bond were no longer detectable, thus indicating their quantitative epoxidation.

**Figure 3:**
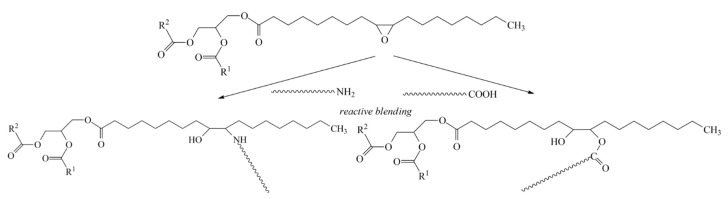
Scheme of reaction between EWVO and terminal groups of PA6.

Due to the presence of the same functional groups, the compatibilization mechanism is expected to be similar to the one reported for Lotader [4]: EWVO is a CP because its epoxy groups are able to react with both the amine and carboxylic terminal groups of PA6 (Figure 3). This reaction takes place in situ, during the HDPE and PA6 blending, and leads to the formation of an oil-terminated polyamide. The latter is the final compatibilizer because it is supposed to migrate to the HDPE/PA interface and improve the interfacial adhesion between the two polymers. The polyamide main chain guarantees suitable compatibility with PA6, while the terminal low polar fatty oil moieties should be more compatible with the polyolefin. Analogously, the Lotader is a CP because after reacting with the PA6 terminal groups, it forms a copolymer having a polyolefin main chain grafted with PA6. The main difference between the two precursors is provided by the molecular weight of the polyolefin-like segment, which is much higher in the case of Lotader.

### 3.3. Blends

**Figure 4:**
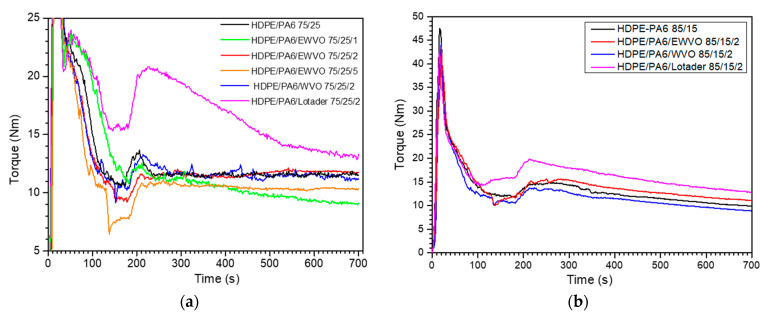
Torque as a function of the mixing time for blends having HDPE as a matrix: (**a**) blends with weight ratio HDPE/PA6 75/25; (**b**) blends with weight ratio HDPE/PA6 85/15.

Figure 4 shows the curves of the torque versus the mixing time for the binary and ternary blends with HDPE/PA6 75/25 (Figure 4a) and 85/15 (Figure 4b) weight ratios. In both cases, the HDPE/PA6/Lotader blends showed higher melt viscosity, thus confirming its ability to strongly influence the interfacial adhesion between the components of the blend [4]. In contrast, the addition of 2 phr of WVO to the corresponding binary blend seems to have a negligible effect on the melt viscosity. However, it is important to highlight that in the case of WVO, the temperature reached during the mixing was lower than that recorded for the other blends (Table 4). All the blendings were conducted with the same temperature profile of the Brabender chamber (set at 235 °C), which may result in different temperatures of the molten material due to the mechanical stress. Therefore, the recorded torque is affected by both the degree of compatibilization and the temperature of the blend. As reported in Table 4, in the case of HDPE/PA6 75/25, the temperature recorded after 700 s of mixing was about 10 °C higher than that of HDPE/PA6/WVO 75/25/2 (245 vs. 234.6 °C). This suggests that WVO had a plasticizing or lubricating effect that reduced the internal friction. In the case of EWVO, the mixing temperature varied depending on the quantity in the blend. If compared to the uncompatibilized blend, the addition of 2 phr of EWVO caused a slight increase in the torque (Figure 4a) and a decrease in the temperature. On the contrary, when EWVO is present in the amount of 5 or 1 phr, both the temperature and the torque are lower than those of HDPE/PA6 72/25. This result suggests that 1 phr led to a lower degree of compatibilization than 2 phr, while, when 5 phr was added, an excess of unreacted molecules of EWVO remained as a low-molecular-weight component with a plasticizing-lubricating effect.

The above-described effects of WVO and EWVO were also clearly visible in the case of blends with an 85/15 HDPE/PA6 weight ratio (Figure 4b).

**Figure 5:**
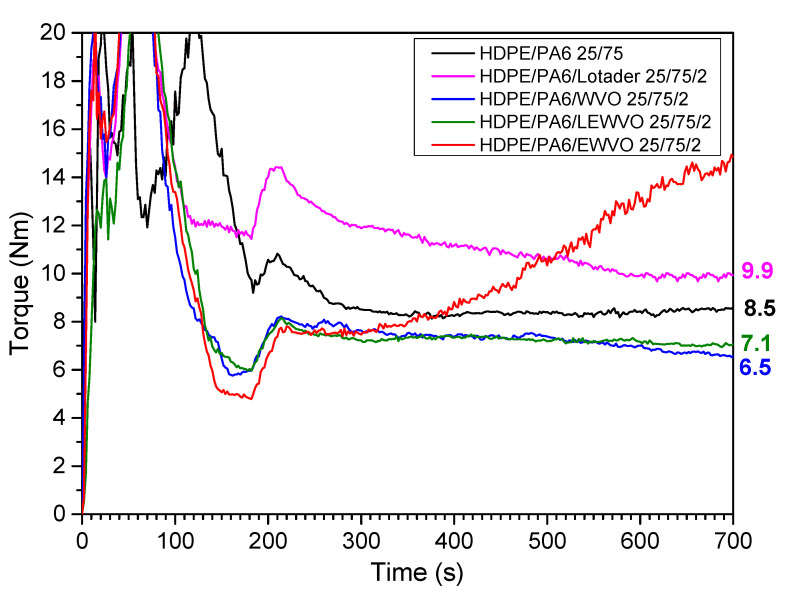
Torque as a function of the mixing time for blends having PA6 as a matrix (blends with HDPE/PA6 weight ratio of 25/75).

In the case of blends with a high content of polyamide, Lotader and WVO had effects that parallel those observed in the blends having HDPE as a matrix. HDPE/PA6/Lotader 25/75/2 and HDPE/PA6/WVO 25/75/2 showed a higher and lower viscosity than HDPE/PA6 25/75, respectively (Figure 5), thus confirming the compatibilizing effect of Lotader and the plasticizing/lubricating effect of WVO. In contrast, the HDPE/PA6/EWVO 25/75/2 sample showed a different trend of the applied torque during blending. The torque failed to level at a constant value and kept increasing during the whole mixing, and the final product was a highly crosslinked material that could not be further processed and characterized. The risk of crosslinking during the reaction between Lotader and PA chains was previously observed by Chiono et al. [4].

Being PA6, a linear polymer with two terminal functional groups, the formation of a three-dimensional crosslinked network is possible only if the CP has more than two functionalities per molecule. Since both Lotader (estimated much more than 2 by Chiono et al. [4]) and the EWVO used in this work (an average of about 3.2 epoxy rings per mol of triglyceride) satisfy this condition, to understand their different behavior, it is necessary to evaluate the total epoxy ring content of the two CPs.

In 75 g of PA6, there are 0.052 mol of (NH_2_ and COOH) groups, while in 2 g of EWVO and Lotader, there are 0.0075 and 0.0017 mol of epoxy rings, respectively. Therefore, in an HDPE/PA6/CP blend, the ratio among CP and PA6 functional groups is equal to 0.144 (EWVO) and 0.0327 (Lotader). In the case of Lotader, there is a very high excess of PA6 functionality with respect to the stoichiometric ratio. Therefore, it is highly probable that a polyamide chain reacts with only one of its terminal groups. In other words, the reaction is more likely expected to form the grafted copolymer. On the contrary, when the oil is involved, the total amount of epoxy rings is more than quadrupled, and the probability that a polyamide chain may react on both sides is much higher. In this case, the chain forms a bridge among oil molecules and contributes to the formation of the network. Therefore, in order to verify this hypothesis, a new CP (named LEWVO) with a lower degree of substitution of C=C with oxirane rings (around 30% instead of 100%) was prepared. With this oil, the total amount of epoxy rings is comparable to that of Lotader, and, as expected, the blend HDPE/PA6/LEWVO 25/75/2 did not crosslink during the mixing, thus confirming the importance of the molar ratio between the functional groups involved in the compatibilization reaction. A preliminary morphological analysis not reported here indicated an improvement in the interfacial adhesion between the two polymers due to LEWVO. Nevertheless, this study is focused on the 100% epoxidized vegetable oil. Therefore, only blends with polyolefin matrix were subjected to further investigation.

**Figure 6:**
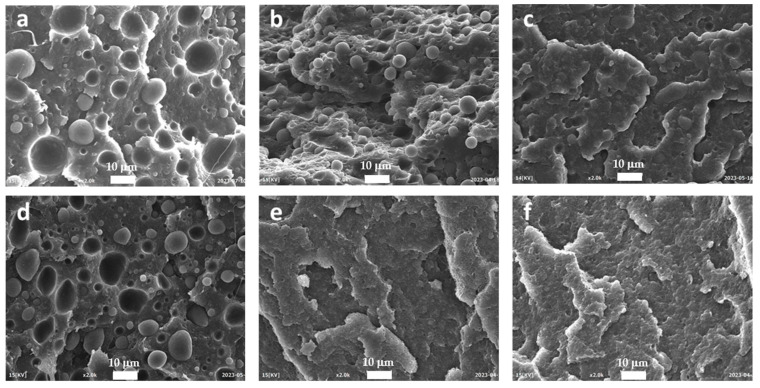
SEM micrographs at 2000× of (**a**) HDPE/PA6 75/25, (**b**) WVO 75/25/2, (**c**) Lotader 75/25/2, (**d**) EWVO 75/25/1, (**e**) EWVO 75/25/2, and (**f**) EWVO 75/25/5.

**Figure 7:**
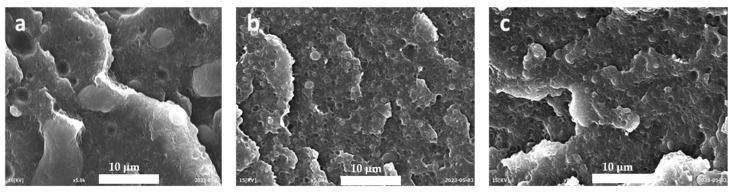
SEM micrographs at 5000× of HDPE/PA6 75/25 with (**a**) 2 phr of Lotader, (**b**) 2 phr of EWVO, and (**c**) 5 phr of EWVO.

**Figure 8:**
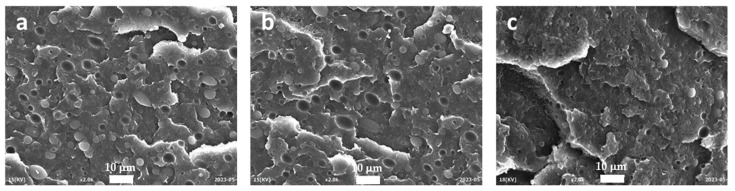
SEM micrographs at 2000× of (**a**) HDPE/PA6 85/15, (**b**) WVO 85/15/2, and (**c**) EWVO 85/15/2.

The effectiveness of EWVO as a compatibilizer is clearly evident by observing the SEM analysis results reported in Figure 6, Figure 7 and Figure 8. As can be noted, the micrograph of HDPE/PA6 75/25 (Figure 6a) showed a biphasic morphology with PA6 droplets, having a wide size distribution, dispersed in the HDPE matrix. Analogous images were obtained when 2 phr of WVO were added to the blend (Figure 6b). However, in this case, the particles seemed to be almost perfectly spherically shaped, thus suggesting even an increase in the interfacial tension between the two phases. A completely different morphology was obtained with the use of Lotader (Figure 6c): the particles were smaller and remained embedded in the HDPE matrix. Figure 6d shows that 1 phr of EWVO was not sufficient to obtain a suitable compatibilization. However, it can be noted that the PA6 droplets showed marked deformation if compared to the spherical shape of the blend with WVO. This is the sign of an increased adhesion between the two phases. Indeed, a morphology similar to that with Lotader was obtained in the HDPE/PA6/EWVO blends with higher oil content (Figure 6e,f), thus confirming its ability to locate at the interface and improve the interfacial adhesion. Moreover, it is interesting to note that the addition of EWVO led to PA6 domains smaller than those obtained with Lotader, as can be clearly seen in the micrographs with higher magnification, reported in Figure 7.

The images obtained with HDPE/PA6 85/15 weight ratio are qualitatively similar to those of the 75/25 blends (Figure 8), thus confirming the effectiveness of EWVO as a CP also in the case of HDPE/PA6 85/15 weight ratio. As for the previous case, the presence of WVO did not affect the interaction between the HDPE matrix and the PA6 domains.

**Figure 9:**
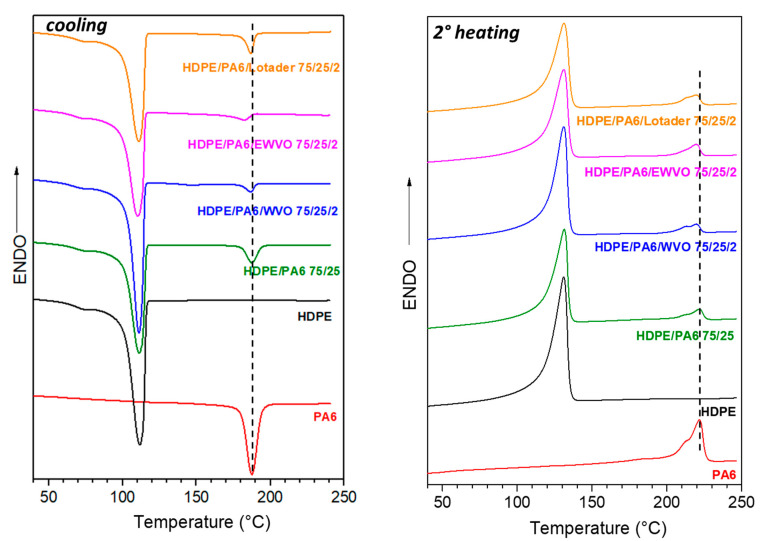
DSC thermograms of pure HDPE, PA6, and their binary and ternary blends with an HDPE/PA6 weight ratio of 75/25: (**left**) cooling and (**right**) second heating run. The dashed lines indicate the crystallization (left) and melting (right) temperatures of pure PA6.

SEM results are consistent with the DSC characterization. The thermograms of the cooling run and the second heating were recorded to evaluate the crystallization and melting behavior, respectively. Figure 9 reports the graphs of the pure components, HDPE and PA6, and the blends with a weight ratio of HDPE/PA6 75/25. The thermal data, that is, the temperatures and enthalpies of crystallization (T_c_, ΔH_c_) and the temperatures and enthalpies of melting (T_m_, ΔH_m_), are reported in Table 5.

The pure HDPE showed a crystallization peak with the maximum at 111.7 °C, while PA6 crystallized at 187.1 °C. The DSC trace of the binary blend HDPE/PA6 75/25 showed two peaks. As can be observed in Table 5, the temperatures and the enthalpies corresponded to the pure polymers’ phases, thus indicating that no interactions occurred between the polyolefin matrix and polyamide domains. On the contrary, the presence of 2 phr of Lotader reduced the crystallinity of the PA6 phase. This result, together with the morphology, confirmed the strong interactions ascribed to the compatibilizing effect [3]. The same effect was even more enhanced in the case of HDPE/PA6/EWVO 75/25/2, where, besides a reduction in the enthalpy values, the crystallization of PA6 occurred in a wider range of temperatures, and the maximum was at 182.9 °C, a value lower than that of pure PA6. It can be noted that the sample containing 2 phr of WVO showed a reduced crystallinity, as the other ternary blends. However, in this case, there are no interactions between the two phases, as shown by the morphological analysis. Therefore, it is reasonable to suppose that the WVO molecules hindered the organization of the PA chains, reducing the crystallinity of the material.

In the case of HDPE/PA6 85/15 blends, the particles of PA6 were highly dispersed in the matrix and crystallized in a wide range of temperatures. Therefore, the peak associated with the crystallization of the polyamide phase was not detectable in the DSC traces of the cooling run (not reported here). Furthermore, the melting of the PA6 crystals was visible in the thermograms of the second heating, and the corresponding enthalpy values suggested a slight reduction in the crystallinity.

**Figure 10:**
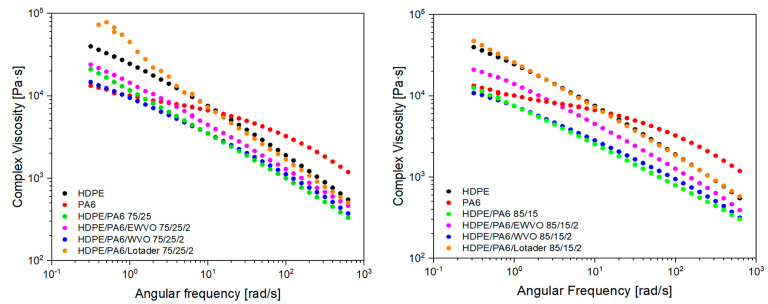
Comparison of rheological behavior of pure HDPE, PA6, and blends with a HDPE/PA6 weight ratio of (**left**) 75/25 and (**right**) 85/15.

The rheological behavior of the blends was also evaluated because it gives interesting information for the processing of the material in a melt state, such as in extrusion or injection molding. The complex viscosities of the pure polymers, binary and ternary blends with a weight ratio of HDPE/PA6 75/25 and 85/15 are reported as a function of angular frequency in Figure 10. The analysis was carried out in a wide range of frequencies; however, the transformation processes are usually performed at high temperatures that correspond to low frequencies. Therefore, we focused our attention on the left side of the curves (from 0.1 to 10 rad/s). In this range, the behavior is in agreement with the torque measured during the reactive blending. In fact, the addition of 2 phr of Lotader or EWVO increased the viscosity of the blend, as well as 2 phr of EWVO, thus confirming a higher level of interactions between the polyolefin and polyamide phases. On the contrary, the presence of 2 phr of the unmodified oil, WVO, reduced the viscosity of the blend according to the plasticizing/lubricating effect recorded during the blending.

**Figure 11:**
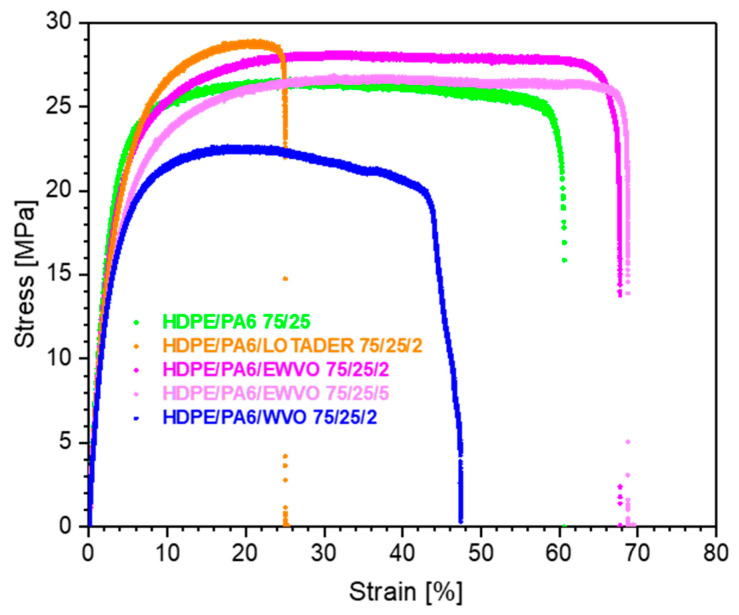
Indicative stress–strain curves of HDPE/PA6 75/25 samples.

**Figure 12:**
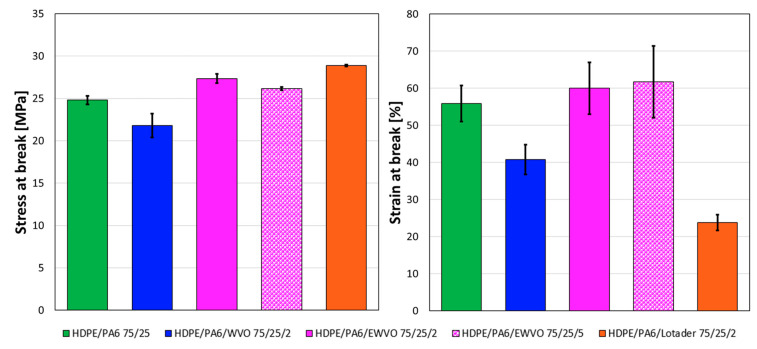
Stress (**left**) and strain (**right**) at break of HDPE/PA6 75/25 samples.

In Figure 11, indicative stress–strain curves of the different samples are reported. Elastic modulus, stress at break, and elongation at break of blends with a weight ratio HDPE/PA6 of 75/25 are reported in Table 6. Blend HDPE/PA6/WVO 75/25/2 showed reduced mechanical performance than the binary blend, suggesting that the presence of WVO molecules in the material had a negative effect on the interfacial adhesion between the matrix and the dispersed particles. On the contrary, 2 phr of Lotader increased the stress at the break at the expense of the ductility of the material. Again, this can be related to the increase in the interfacial adhesion between the two polymeric phases. Interesting results were obtained with EWVO, which yielded increased stress at break, if compared to the uncompatibilized blend, and an elongation at break significantly higher than that of the sample with Lotader (this can be better appreciated in Figure 12). Compared to the HDPE/PA6/Lotader blend, the different mechanical behavior of HDPE/PA6/EWVO blends can be ascribed to the finer size of the dispersed phase [40], as shown in Figure 7 by SEM micrographs. Moreover, in the blend with 5 phr of EWVO, a lubricating effect of the unreacted molecules may contribute to an increase in the strain at break.

## 4. Conclusions

In this study, an epoxidized vegetable oil (EWVO) derived from a waste cooking oil was investigated as a potential green compatibilizer precursor (CP) for blends of HDPE and PA6. The EWVO was synthesized by epoxidation reaction using an ion-exchange resin as a heterogenous catalyst. Blends with different weight ratios of HDPE/PA6 (75/25, 85/15, 25/75) and amounts of EWVO (1, 2, 5 phr) were prepared in order to evaluate the effectiveness of EWVO in the case of polyolefin and polyamide matrix. A commercial CP (Lotader) with epoxy functional groups, such as EWVO, and the unmodified oil (WVO) were also used for comparison. Epoxy rings of EWVO showed their ability to react with the amine and carboxylic groups during the reactive blending, as Lotader. However, when the polyamide constitutes the continuous phase, the covalent bonding may result in a three-dimensional network, leading to a highly crosslinked material. In this case, a careful evaluation of the concentration of epoxy functionalities is necessary to avoid the problem. On the contrary, the use of EWVO in blends with HDPE/PA6 75/25 and 85/15 ratios led to materials with better properties as compared to blends prepared without a CP. In this case, the morphological analysis showed that 2 phr of EWVO is the minimum quantity to obtain an interesting compatibilizing effect. The increased interactions between the two polymers were confirmed by the reduced crystallinity of the PA dispersed phase and by both rheological and tensile tests. It is worth noting that, conversely to Lotader, EWVO improved all the mechanical properties, increasing the elastic modulus and the stress at break without a reduction in the elongation at break. This can be attributed to the smaller size of the dispersed particles of the blends obtained with the addition of EWVO. In conclusion, all these results indicated that EWVO has a suitable potential as an alternative bio-based CP in HDPE/PA blends with high HDPE content.

## Figures and Tables

**Table 1:** Assignment of proton signals.

Signal	Group	#Proton
*a*	Olefinic protons in the glyceryl group	2
*b*	Methine proton in the glyceryl group	2
*c*	The four methylene protons in the glyceryl group	4
*d*	Divinyl methylene protons	2
*e*	The six α-methylene protons adjacent to the carbonyl group	2
*f*	Allyl methylene protons	2
*g*	The six β-methylene protons from carbonyl carbon	2
*h*	Methylene protons on saturated carbon atoms	2
*i* + *j*	The nine terminal methyl protons	3

**Table 2:** List of prepared blends.

Sample Name	HDPE (wt.%)	PA6 (wt.%)	CP	
			Name	phr
HDPE	100	-		
PA6	-	100		
HDPE/PA6 75/25	75	25	-	-
HDPE/PA6/EWVO 75/25/1	75	25	EWVO	1
HDPE/PA6/EWVO 75/25/2	75	25	EWVO	2
HDPE/PA6/EWVO 75/25/5	75	25	EWVO	5
HDPE/PA6/WVO 75/25/2	75	25	WVO	2
HDPE/PA6/Lotader 75/25/2	75	25	Lotader	2
HDPE/PA6 85/15	85	15	-	-
HDPE/PA6/EWVO 85/15/2	85	15	EWVO	2
HDPE/PA6/WVO 85/15/2	85	15	WVO	2
HDPE/PA6/Lotader 85/15/2	85	15	Lotader	2
HDPE/PA6 25/75	25	75	-	-
HDPE/PA6/EWVO 25/75/2	25	75	EWVO	2
HDPE/PA6/WVO 25/75/2	25	75	WVO	2
HDPE/PA6/Lotader 25/75/2	25	75	Lotader	2

**Table 3:** Intensity of the proton’s signals.

Signal	*a + b*	*c*	*d*	*e*	*f*	*g*	*h*	*i + j*
Intensity	2.46	1.32	0.61	2.00	3.20	2.10	18.81	2.96

**Table 4:** Torque and temperature values at 700 s of blending.

Blend	Torque (Nm)	Temperature (°C)
HDPE/PA6 75/25	10.84	245.0
HDPE/PA6/EWVO 75/25/1	9.1	241.5
HDPE/PA6/EWVO 75/25/2	11.7	242.3
HDPE/PA6/EWVO 75/25/5	10.3	242.0
HDPE/PA6/WVO 75/25/2	11.2	234.6
HDPE/PA6/Lotader 75/25/2	13.3	245.4
HDPE/PA6 85/15	9.85	245.0
HDPE/PA6/EWVO 85/15/2	11.13	247.7
HDPE/PA6/WVO 85/15/2	8.84	245.8
HDPE/PA6/Lotader 85/15/2	12.7	251.3

**Table 5:** Thermal properties of HDPE, PA6 blends with HDPE/PA6 weight ratios of 75/25 and 85/15.

Sample	HDPE Phase				PA6 Phase			
	T_c_ (°C)	ΔH_c_ (J/g)	T_m_ (°C)	ΔH_m_ (J/g)	T_c_ (°C)	ΔH_c_ (J/g)	T_m_ (°C)	ΔH_m_ (J/g)
HDPE	111.7	191.9	131.1	182.0	-	-	-	-
PA6	-	-	-	-	187.1	67.9	221.7	67.5
HDPE/PA6 75/25	111.6	190.8	130.4	180.0	187.5	64.5	219.6	67.0
HDPE/PA6/Lotader 75/25/2	111.3	189.0	131.2	186.0	186.7	48.3	219.7	52.6
HDPE/PA6/EWVO 75/25/2	110.4	182.5	131.1	188.2	182.9	31.1	219.7	54.0
HDPE/PA6/WVO 75/25/2	111.1	194.8	131.1	209.0	186.7	21.6	212.4	47.8
HDPE/PA6 85/15	110.6	192.6	130.7	194.9	-	-	219.4	59.7
HDPE/PA6/Lotader 85/15/2	111.5	190.0	130.5	192.3	-	-	218.1	65.7
HDPE/PA6/EWVO 85/15/2	109.2	182.5	131.6	181.3	-	-	219.1	62.2
HDPE/PA6/WVO 85/15/2	110.1	183.7	130.8	187.2	-	-	219.3	61.9

**Table 6:** Mechanical properties.

Blend	Elastic Modulus (GPa)	Stress at Break (MPa)	Elongation at Break (%)
HDPE	1.29 ± 0.23	26.1 ± 0.2	82 ± 4
PA6	2.69 ± 0.20	67.0 ± 4.8	225 ± 3
HDPE/PA6 75/25	1.18 ± 0.13	24.8 ± 0.5	56 ± 5
HDPE/PA6/WVO 75/25/2	1.08 ± 0.14	21.8 ± 1.8	41 ± 4
HDPE/PA6/EWVO 75/25/2	1.26 ± 0.08	27.3 ± 0.5	60 ± 7
HDPE/PA6/EWVO 75/25/5	1.04 ± 0.17	26.2 ± 0.2	62 ± 9
HDPE/PA6/Lotader 75/25/2	1.13 ± 0.11	28.9 ± 0.1	24 ± 2

## Data Availability

All raw data are available on request.
